# Non-Infectious Diseases Compatible With COVID-19 Pneumonia

**DOI:** 10.7759/cureus.9989

**Published:** 2020-08-24

**Authors:** Burcu Baran Ketencioğlu, Fatma Yiğit, Mohammed Almadqa, Nuri Tutar, İnsu Yılmaz

**Affiliations:** 1 Respiratory Medicine, School of Medicine, Erciyes University, Kayseri, TUR

**Keywords:** coronavirus disease 2019 (covid-19), rt-pcr (real time - reverse transcription polymerase chain reaction), ct (computed tomography) imaging, peripheral eosinophilia

## Abstract

While the definitive diagnosis of the coronavirus disease 19 (COVID-19) is mainly made by the polymerase chain reaction (PCR), some PCR-negative cases are diagnosed typically by a computed tomography (CT) scan's radiology. However, there are many different infectious and non-infectious diseases that have radiology like COVID-19. We are presenting a case of a patient having symptoms and a CT scan radiology comparable to that of COVID-19 and also having eosinophilia. The patient was initially diagnosed and treated as a COVID-19 patient. The patient stated that she had always complained of having dyspnea and cough, but it had increased even more in the past few days. Her thorax CT revealed bilateral ground-glass opacities with upper lobe predominance, which was reported as highly compatible with COVID-19 by radiologists. COVID-19 PCR result was negative twice. In laboratory results, eosinophil count was 2,850/mm^3^ and total Ig was 768 IU/mL. However, when the laboratory values and the radiological findings were combined with the patient’s history, COVID-19 was excluded and the chronic eosinophilic pneumonia was accepted as a diagnosis. Clinicians more focused on COVID-19 while questioning the patients and while evaluating the laboratory and the radiological findings make it easier to miss other infectious and non-infectious diseases. Assessing the complete blood count result, focusing on the lymphocyte value, also makes it easy to skip eosinophilia.

## Introduction

The new coronavirus disease caused by severe acute respiratory syndrome coronavirus 2 (SARS COV-2) started in Wuhan, China, and became a global pandemic. It was named officially COVID-19 by the World Health Organization on February 11, 2020 [1]. The disease is mainly considered to be airborne, where the respiratory symptoms are mostly accompanied by fever. In addition to thorax computed tomography (CT), the SARS-CoV-2-specific real-time reverse transcription-polymerase chain reaction (rRT-PCR) as a gold standard test using a swab from the nasopharyngeal and the oropharyngeal is the primary diagnostic tool. Besides, the detection of the nucleic acid was easily affected by many factors, which include insufficient cellular materials and, clinically, the improper extraction of the nucleic acid materials, which made the rRT-PCR more susceptible to false-negative results [2]. In the COVID-19 suspected cases which had a negative PCR test, the diagnosis was made by thorax CT. In previous studies, it was reported that the CT was more sensitive than the PCR [3]. In the pandemic period, even if we are more focused on the COVID-19 disease, we should not forget that other conditions still continue. Some infectious (viral Infections, bacterial infections, *Pneumocystis jirovecii* pneumonia) and non-infectious diseases (interstitial lung disease, vasculitis, etc.) can have respiratory symptoms and CT findings, which are similar to that of COVID-19 [4]. That is why the comprehensive evaluation of the patients in the laboratory, clinically and radiologically, is essential in these pandemic days as well. In this report, we present a patient who had clinical and radiological findings that were compatible with COVID-19 but were diagnosed to be chronic eosinophilic pneumonia (CEP).

## Case presentation

A 40-year-old female patient was admitted to the pandemic outpatient clinic of the university with a two-day history of cough, dyspnea, and abdominal pain. The patient was routinely followed at the chest diseases outpatient clinic for a 13-year history of asthma and was committed to the use of high-dosage long-acting beta-agonist and inhaled corticosteroids. The patient stated that she had always complained of having dyspnea and cough, but it had increased even more in the past few days. The patient had no history of direct contact with a COVID-19 positive patient and had no history of travel abroad.

**Figure 1:**
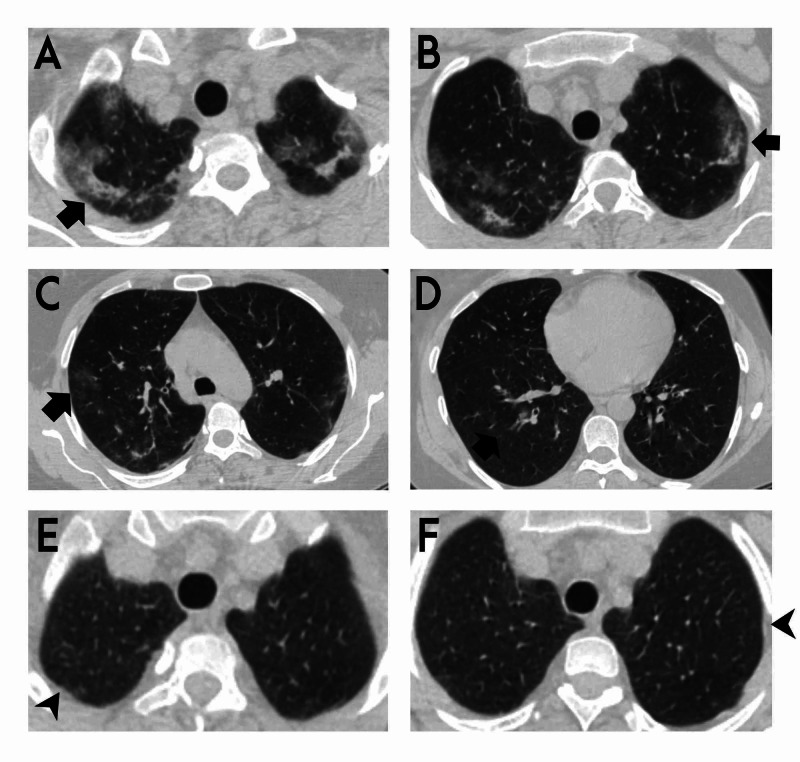
Thorax CT sections of the patient (A-D) Bilateral ground-glass opacities with peripheral and upper zone predominance, as shown on thorax CT by arrows. (E and F) After four weeks of treatment with oral prednisone, lesions disappeared in control thorax CT, as shown by arrowheads.

At hospital admission her vital signs were as follows: temperature of 36.5 C, blood pressure of 120/70 mmHg, pulse rate of 86 beats/min, respiratory rate of 22 breaths/min, and blood oxygen saturation (SpO2) of 96% in room air. On the respiratory system examination, the breathing sounds increased in both lungs and the expiration period was prolonged. No other abnormalities were found during the systemic examination. Her thorax CT revealed bilateral ground-glass opacities with upper lobe predominance, which was reported as highly compatible with COVID-19 by radiologists (Figures 1A-1D). The patient was hospitalized and was started on hydroxychloroquine and azithromycin treatment. According to laboratory results, C-reactive protein (CRP) was 7.75 mg/L, hemoglobin was 12.2 g/dL, and white blood cell count (WBC) was 7,660/mm^3^ with 2,850/mm^3^ eosinophils. The liver and kidney function tests were normal. COVID-19 rRT-PCR result was negative twice. No clinical improvement was detected on the third day of hospitalization, and the patient was investigated for the etiology of eosinophilia. Immunoglobulin E (IgE) level was 768 IU/mL, vitamin B12 level was 215 pg/mL and stool parasitology was negative. Antinuclear antibody, anti-cytoplasmic antibody profile, and genetic markers were studied for eosinophilic granulomatosis with polyangiitis (EGPA) and hypereosinophilic syndrome (HEP). The results were negative. Bronchoscopy was not performed because the patient was suspected of having the COVID-19 infection. Finally, after excluding other causes of eosinophilia (EGPA, allergic bronchopulmonary aspergillosis, HEP, drug-related eosinophilia, and Loeffler’s pneumonia), radiological findings, two negative COVID-19 PCR results, having a history of asthma, and being symptomatic for a long time despite the usage of inhaler treatments, COVID-19 infection was excluded and the diagnosis of CEP was prioritized. Oral prednisone treatment was started with a dose of 0.5 mg/kg per day. The patient responded to steroid treatment dramatically. Cough, dyspnea, and constitutional symptoms had decreased significantly, and the patient was discharged on her fifth day of hospitalization. After four weeks of treatment with prednisone, the patient's symptoms had totally improved. The control eosinophil value was 0 x 10^3^/uL and the IgE value was 190 IU/mL. At thorax CT control, the lesions had almost completely disappeared (Figures 1E, 1F).

## Discussion

Our case is the first CEP case in the literature that was misdiagnosed as COVID-19. CEP is an idiopathic condition characterized by the alveoli being filled with inflammatory, eosinophil-rich infiltrate. Classically on imaging, it appears as consolidative opacities with upper zone and peripheral predominance [5]. More than half of the CEP patients have accompanying allergic diseases such as bronchial asthma, atopic dermatitis, and allergic rhinitis. The onset of allergic disease may be before or after the onset of CEP [6]. In CEP patients, cough, shortness of breath, and constitutional symptoms are the main symptoms. The diagnosis of CEP is classically based on the combination of clinical presentation, chest imaging showing predominantly peripheral or pleural-based, middle to upper lung zone opacities, and a bronchoalveolar lavage showing eosinophilia ≥ 40% (at least ≥25% or peripheral blood eosinophils ≥1,000/mm^3^). Other potential causes of eosinophilic pneumonia, such as infection, drugs, and vasculitis, should be excluded [7]. Blood eosinophils of nearly 30% and increased IgE levels of more than 500 IU are the critical findings for the diagnosis of CEP. Slightly increased C-reactive protein and WBC can also be seen [8-11]. This patient was admitted to the hospital with respiratory symptoms, and a suspicious case was accepted for COVID-19 infection due to pandemic.

Although studies [12,13] have shown that eosinopenia is generally related to COVID-19 infection, this patient had 37.2% of blood eosinophilia. In the patient’s radiology, bilateral, especially in the subpleural area, ground-glass densities were present and accepted by the expert radiologist as typical findings compatible with COVID-19. However, while lower lobe dominance is expected in coronavirus disease, in the patient's thorax CT scan upper lobe involvement was dominant. Although two PCR test results were negative, the patient was accepted as COVID-19 and was isolated in order to prevent transmission risk and disease progression. When the laboratory values and the radiological findings were combined with the patient’s history, COVID-19 was excluded, and the CEP was accepted as a diagnosis. The clinical, laboratory, and radiological response of the patient to systemic steroid treatment supported the diagnosis of CEP.

## Conclusions

Clinicians being more focused on COVID-19 while questioning the patients and evaluating the laboratory and the radiological findings make it easier to miss other infectious and non-infectious diseases. Assessing the complete blood count result, focusing on the lymphocyte value, also make it easy to skip eosinophilia. Therefore, it is important to evaluate these patients with the clinicians of the infectious diseases, chest diseases, and radiology departments in a multidisciplinary way.
